# Effect of robotic-assisted gait training on objective biomechanical measures of gait in persons post-stroke: a systematic review and meta-analysis

**DOI:** 10.1186/s12984-021-00857-9

**Published:** 2021-04-16

**Authors:** Heidi Nedergård, Ashokan Arumugam, Marlene Sandlund, Anna Bråndal, Charlotte K. Häger

**Affiliations:** 1 Department of Community Medicine and Rehabilitation, Physiotherapy, Umeå University, Umeå, Sweden; 2 Department of Physiotherapy, College of Health Sciences, University of Sharjah, Sharjah, United Arab Emirates

**Keywords:** Cerebrovascular accident, Powered exoskeleton, Walk, Rehabilitation, Literature synthesis

## Abstract

**Background:**

Robotic-Assisted Gait Training (RAGT) may enable high-intensive and task-specific gait training post-stroke. The effect of RAGT on gait movement patterns has however not been comprehensively reviewed. The purpose of this review was to summarize the evidence for potentially superior effects of RAGT on biomechanical measures of gait post-stroke when compared with non-robotic gait training alone.

**Methods:**

Nine databases were searched using database-specific search terms from their inception until January 2021. We included randomized controlled trials investigating the effects of RAGT (e.g., using exoskeletons or end-effectors) on spatiotemporal, kinematic and kinetic parameters among adults suffering from any stage of stroke. Screening, data extraction and judgement of risk of bias (using the Cochrane Risk of bias 2 tool) were performed by 2–3 independent reviewers. The Grading of Recommendations Assessment Development and Evaluation (GRADE) criteria were used to evaluate the certainty of evidence for the biomechanical gait measures of interest.

**Results:**

Thirteen studies including a total of 412 individuals (mean age: 52–69 years; 264 males) met eligibility criteria and were included. RAGT was employed either as monotherapy or in combination with other therapies in a subacute or chronic phase post-stroke. The included studies showed a high risk of bias (n = 6), some concerns (n = 6) or a low risk of bias (n = 1). Meta-analyses using a random-effects model for gait speed, cadence, step length (non-affected side) and spatial asymmetry revealed no significant differences between the RAGT and comparator groups, while stride length (mean difference [MD] 2.86 cm), step length (affected side; MD 2.67 cm) and temporal asymmetry calculated in ratio-values (MD 0.09) improved slightly more in the RAGT groups. There were serious weaknesses with almost all GRADE domains (risk of bias, consistency, directness, or precision of the findings) for the included outcome measures (spatiotemporal and kinematic gait parameters). Kinetic parameters were not reported at all.

**Conclusion:**

There were few relevant studies and the review synthesis revealed a very low certainty in current evidence for employing RAGT to improve gait biomechanics post-stroke. Further high-quality, robust clinical trials on RAGT that complement clinical data with biomechanical data are thus warranted to disentangle the potential effects of such interventions on gait biomechanics post-stroke.

**Supplementary Information:**

The online version contains supplementary material available at 10.1186/s12984-021-00857-9.

## Background

Technology-assisted interventions to enhance gait rehabilitation post-stroke are highly interesting from a clinical perspective. Robotic-assisted gait training (RAGT) employs electromechanical devices that assist stepping cycles by supporting body weight while automatizing the gait process through support and facilitation of movement in one or several lower limb joints. RAGT is suggested to be less energy-consuming and cardiorespiratory demanding when compared with walking without a robot [1]. Implementing RAGT may thus enable higher intensities and longer, task-specific training sessions when compared with non-robotic gait training.

Various forms of robotic devices are commercially available and they are commonly categorized according to the support they apply [2]. Treadmill-based RAGT (t-RAGT) is most commonly used in combination with body weight support [3]. This is either performed with end-effector robots that drive two footplates, simulating the phases of the gait, or with exoskeleton orthoses that move the lower body extremity joints in coordination with the phases of gait. Overground RAGT (o-RAGT) is provided by wearable powered exoskeletons that allow a person to walk overground on hard and flat surfaces [4], supposedly enabling the user to experience increased proprioceptive input when compared with the stationary treadmill training [5].

Earlier reviews revealed that RAGT, together with conventional physiotherapy, might have a slightly better or similar positive effect on gait speed and ambulation when compared with conventional gait training alone [6–16]. However, the need for a broadened perspective in the evaluation of gait ability after RAGT post-stroke has been highlighted [13, 15, 17, 18]. The International Classification of Functioning, Disability and Health (ICF), advocated by the World Health Organization, is a classification system widely used in clinical practice [19]. It is a foundation for understanding the patient’s personal and environmental resources and limitations, hence also used when evaluating rehabilitation effects from different perspectives. The classification system identifies three domains of a health condition: (1) body function (physiological and psychological) and structure (related to organs, limbs, etc.), (2) activity (related to the execution of a task, and (3) participation (related to involvement in a real-life situation). Although the domains are interrelated, measurements of all domains and contextual factors are necessary to describe a person’s condition from a holistic point of view. In a 2013 review, Geroin and colleagues [20] emphasize that a comprehensive post-intervention evaluation of RAGT, such as that of any other intervention, should use outcome measures that include all domains of the ICF. In general, tests that evaluate walking ability post-stroke address activity limitations alone (6 min Walk Test, Timed Up and Go, Functional Ambulation Category). These tests might fail to identify restrictions related to the domain of body function and structure since they do not investigate specific gait characteristics, such as coordination, muscle power, joint mobility or extremity positions during gait. In persons post-stroke, gains in walking ability following rehabilitation may be considered a result of the restitution of underlying impairments. However, improvements in activity measures could also partly be explained by an adaptation of non-optimal movement strategies that compensate for existing deficits [21, 22]. A paradigm shift has occurred in the research area of gait rehabilitation post-stroke [23], claiming that rehabilitation methods that stimulate the nervous system's ability to recover a normalized movement pattern should be preferred before those encouraging compensation for impaired mobility, motor control, and balance. In line with this, the quantitative evaluation of gait quality and movement pattern may allow for differentiation of recovery mechanisms and foster a deeper understanding of the effects of different gait rehabilitation interventions post-stroke [18, 23, 24]. To manage this, various biomechanical variables of temporal (related to time) or spatial (related to distance) information have been applied. These are derived from kinematic (parameters of registered position, motion and/or marker trajectories of interest to describe the locomotion pattern) or kinetic (registered forces that act on the body during movement) measures of gait [24]. A gait-assisting robot aims to replicate a movement pattern that is as close to normal as possible with regards to temporal and spatial parameters. It is also believed to generate more repetitions with regards to the number of steps during one training session as compared with non-robotic gait training. RAGT could thus be assumed to improve gait quality to a greater extent than training without a robot by normalizing the movement pattern and increasing training volume with a carryover effect to when the person is walking without the assisting robot. This review aims to summarize the level of evidence for any potential superior effects of RAGT (with or without a combination of non-robotic training) compared with non-robotic training alone on post-stroke gait movement pattern quantified with objective biomechanical measures.

## Methods

### Design and registration

This review followed a protocol pre-registered in PROSPERO (CRD42020168846). The Preferred Reporting Items for Systematic review and Meta-Analysis (PRISMA) statement was used as a framework to document the objectives, methods and findings of the review [25, 26]. Following PRISMA recommendations, the research question and the eligibility criteria were framed using the PICO approach (representing the patient population (P), the interventions (I), the comparator group (C), and the outcome (O), and the study design chosen [27].

### Eligibility criteria

#### Type of studies

This review included only randomized clinical trials (RCT) that investigated the effects of robotic-assisted gait training using instrumented gait analysis to evaluate gait performance during overground or treadmill walking. All other study designs were excluded.

#### Type of participants

This review included adult participants (≥ 18 years of age) in an acute, subacute or chronic phase post-stroke. The stroke could be due to haemorrhagic or ischemic causes. No restrictions were made regarding the functional ability or gender of the participants with regards to inclusion.

#### Type of intervention and comparator groups

RAGT for gait rehabilitation in either an inpatient or outpatient setting was mandatory for inclusion in this review. RAGT was defined as robotic-assisted gait training using an electromechanical device to assist the stepping cycles during walking. The devices could be either end-effectors or exoskeletons for treadmill gait training or exoskeletons used for overground gait training [3]. Contemporary evidence and recommendations suggest that RAGT should complement, not replace, existing gait rehabilitation and non-robotic physical therapies [3, 13]. We therefore also included studies using a combination of RAGT and other therapies such as conventional physiotherapy training or functional electrical stimulation (FES). All studies were nevertheless required to have at least one comparator group performing active, non-robotic gait rehabilitation post-stroke. Non-weight-bearing interventions that used non-interactive devices for delivering continuous passive motion (e.g., an isokinetic apparatus for passive knee flexion [28]), or devices used for seated or standing lower extremity training (e.g., the MotionMaker™ [29], the Rutgers Ankle [30] or the Active Knee Rehabilitation Orthotic Devices (AKROD) [31]) were excluded.

#### Outcomes of interest

Instrumented gait analysis was to be performed in either a laboratory or a clinical setting using devices that register kinematic or kinetic parameters during walking: a 3-dimensional (3D) or 2-dimensional (2D) motion capture system, an optoelectrical or inertial system, a gait or pressure mat, force shoes, a magnetic or acoustic tracking system, etc. Outcome measures of interest were parameters related to temporal and spatial information based on kinematics and kinetics. Studies that assessed gait biomechanics during RAGT, while wearing the robotic device, or immediately after only a single training session were excluded. Results of biomechanical outcomes measured solely by clinical testing, such as gait speed evaluated with a stopwatch or cadence reported from observations were also excluded.

### Search strategy

One reviewer (HN) performed a systematic search in the following databases: PubMed, Web of Science, EBSCO (Cumulative Index to Nursing and Allied Health Literature [CINAHL], Allied and Complementary Medicine [AMED], Academic Search Premier, Sports Discus), Scopus, ProQuest (Sports Medicine & Education Index) and the Cochrane Central Register of Controlled Trials (CENTRAL). The search was limited to full-text articles published in English from the inception of the databases until the 19th of January 2021.

The full search strategy is provided in detail as an additional file (see Additional file 1).

### Screening process

**Fig. 1:**
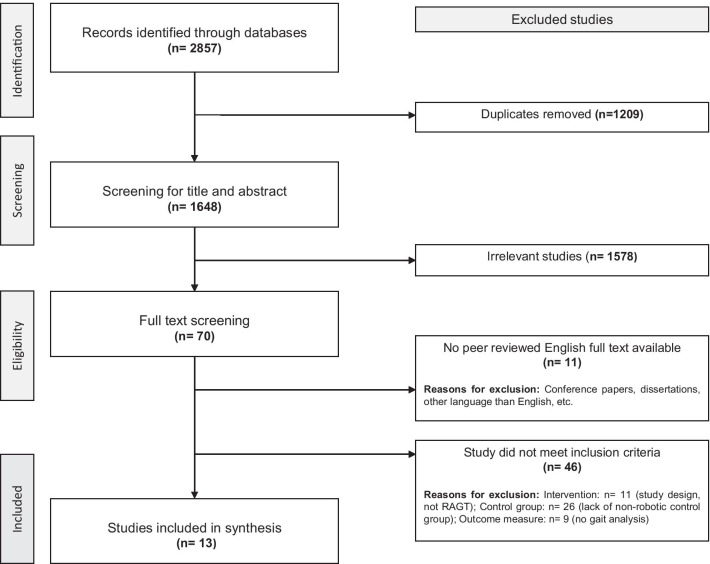
PRISMA flowchart for identification and screening of eligible studies for the current review

The screening process strictly adhered to the ‘a priori’ objective eligibility criteria elaborated in the PROSPERO protocol. Abstracts and titles retrieved in the search of the electronic databases were exported to EndNote X9 and screened independently by two reviewers (HN and AB) to reduce the possibility of rejecting relevant reports (Fig. 1). Only studies that did not clearly match the inclusion criteria were excluded (e.g. populations such as individuals with Parkinson's disease, traumatic brain injury, etc.; study designs such as case studies, cross-sectional studies, etc.; different types of robots, like robots for training the upper extremities, etc.; types of reports such as conference papers, reviews, etc.). All remaining articles advanced to the next step of the screening process and were scrutinized in full-text before potential inclusion. The included articles were divided between two reviewers (HN and AB or MS) for independent data extraction. Risk of bias was independently assessed by the same two reviewers using the Cochrane Risk of Bias 2 (RoB 2) tool [32]. Another reviewer (AA) was available to adjudicate any potential disagreements to help reach a consensus.

A meta-analysis using a random-effects model was performed with Review Manager 5 (Copenhagen: The Nordic Cochrane Centre, Cochrane) when a minimum of three studies with relevant data, adequate homogeneity of population, interventions and chosen outcome measures were available. An I^2^ value > 40% was considered as the threshold for statistical heterogeneity [33]. Subgroup analyses were performed regarding the effects on gait speed and cadence depending on velocity during the assessment (self-selected, SSV, versus fastest possible, FV), type of RAGT employed in the intervention group (t-RAGT versus o-RAGT) and time of publication (studies published 2007–2014 versus studies published 2015–2020). When a study had two intervention groups and one comparator group, the data from the intervention groups were pooled (if their findings were identical) in the synthesis. For instance, one study [34] used two intervention groups performing RAGT. One used additional direct transcranial stimulation during RAGT, while the other group received sham transcranial direct stimulation during the same training. The results in these two groups did not differ significantly and were therefore pooled in the meta-analyses.

In addition to the meta-analyses, a descriptive synthesis was performed for the outcomes where statistical pooling was not possible and findings have been presented in a narrative form with complementing tables. The GRADE (Grading of Assessment, Development and Evaluation) criteria [35] were employed to interpret findings and summarise the levels of evidence for both the pooled and narratively summarised data [36]. The evidence was downgraded from “high certainty” by one level for serious (two levels for very serious) concerns about the risk of bias, indirectness of evidence, the inconsistency of findings, imprecision of effect estimates or potential publication bias across studies.

## Results

### Characteristics of the included studies

Of the 2857 studies retrieved, 13 studies involving a total of 412 participants (264 males, 148 females) were eventually included in this review (Table 1). The mean age of the populations in each study ranged from 52 to 69 years. Sample sizes ranged from 12 to 63 participants and over half of the studies (62%) involved sample sizes of 30 participants or less [34, 37–43]. The participants in three of the studies [43–45] were in a subacute phase (mean time up to 4 months post-stroke), while the rest were in a chronic phase post-stroke (mean time up to 104 months post-stroke). Gait ability for inclusion in each study varied from being independent to needing an assistive device and/or personal assistance for walking. The intervention groups received either RAGT as a monotherapy (n = 10) [34, 37–40, 42, 44–47] or in combination with some kind of non-robotic gait training (n = 3) that was similar to the training received by the comparator group [41, 43, 48]. All studies except one [34] used exoskeletons (Lokomat, GEMS, EKSO, ALEXII, SMA, GEAR) and the majority of these (n = 8) employed t-RAGT with body weight support [34, 37, 38, 40, 41, 43, 44, 47]. One study [39] combined t-RAGT (no body weight support) and o-RAGT. The comparator groups of the included studies received conventional gait training [43–45, 48], overground gait training [34] and/or treadmill gait training with [37, 38, 40–42, 47] or without body weight support [39, 46] (Table 1).

**Table 1:** Descriptive analysis of the included studies organized by name of the first author

Author (year)	*Population* lntervention group				Comparator group					*Intervention*				*Details of assessments*				
	n (male/female)	Age (mean ± SD)	Time since stroke (months)	Drop outs (n)	n (male/female)	Age (mean ± SD)	Time since stroke (months)	Drop outs (n)	Gait ability for inclusion	Device for RAGT	Intervention setting	Comparator group setting	Training intensity	Time for assess- ments	Walking condition for assess-ment	Velo-city during analysis	Gait analysis system	Walking device during gait analysis
Bang 2016	9 (5/4)	54 ± 4	12 ± 3	0	9 (4/5)	54 ± 4	13 ± 3	0	Gait speed > 0,4 m/s; possible independent gait > 10 m	Lokomat	t-RAGT	Treadmill gait training	60 min, 5 d/week, 4 weeks, 20 sessions	Baseline and post training	4,6 m walkway, No info on trials	No info	GAITRite	Not reported
Buesing 2015	25 (17/8)	60 ± 2	85 ± 18	0	25 (16/9)	62 ± 3	65 ± 10	0	Gait speed between 0.4–0.8 m/s; ability to walk 10 m with maximum 1 person assist	Stride Manag-ement Assistance, SMA	o-RAGT	Over ground or treadmill gait training, functional mobility training	45 min, 3 d/week, 6–8 weeks, max 18 sessions	Baseline, midpoint and post training, and at 3 months follow up	5 feet before and after GAITRite, 3 + 3 trials	Self-selected and fastest velocity	GAITRite	Assistive device allowed
Calabró 2018	20 (12/8)	69 ± 4	10 ± 3	0	20 (11/9)	67 ± 6	11 ± 3	0	Functional Ambulatory Categories (FAC) of ≤ 4	EKSO	o-RAGT and over ground gait training	Over ground gait training	45 min + 60 min/d, 5 d/week, 8 weeks, 40 sessions	Baseline and post training	10 m walkway, 2 trials	Self-selected velocity	Accelerometer	Not reported
Geroin 2011	10 (8/2) 10 (6/4)	64 ± 7/64 ± 6	26 ± 6/27 ± 5	0	10 (9/1)	61 ± 6	27 ± 6	0	Ability to walk independently for at least 15 m with the use of walking aids (cane and orthoses)	Gait Trainer (GT1)	(1) t-RAGT with BWS and with transcranial direct current stimulation (2) t-RAGT with BWS and with sham transcranial stimulation	Over ground gait training	50 min/session, 5 d/week, 2 weeks, 10 sessions	Baseline and post training	12 m walkway, 3 trials	Fastest velocity	GAITRite, Bertrec	Orthoses allowed
Hidler 2009	33 (21/12)	60 ± 11	4 ± 2	9*	30 (18/12)	55 ± 9	5 ± 2	*	Ability to ambulate 5 m without physical assistance and a self-selected walking speed between 0.1 to 0.6 m/s	Lokomat	t-RAGT with BWS	Conventional gait training	45 min (90 min), 3 d/week, 24 sessions	At baseline, midpoint and post training, and at a 3 months follow-up	Over ground walkway, No further info	Self-selected velocity	GAITRite or GaitMat	Not reported
Hornby 2008	24 (15/9)	57 ± 10	50 ± 51	4	24 (15/9)	57 ± 11	73 ± 87	10	Required to walk 10 m over ground without physical assistance at speeds 0.8 m/s at their self-selected velocity, assistive device if needed	Lokomat	t-RAGT with BWS	Treadmill gait training with BWS	30 min, 12 sessions	At baseline, post training and at 6-months follow-up	10 m, over ground, > 5 trials	Self-selected and fastest velocity	GaitMat II	No physical assistance, orthoses if needed
Husemann 2007	16 (11/5)	60 ± 13	3 ± 2	3	14 (10/4)	57 ± 11	3 ± 2	1	The patient had to score 1 or less on the functional ambulation classification, indicating a need for personal assistance in ambulation	Lokomat	t-RAGT with BWS + Conventional gait training	Conventional gait training	2 × 30 min/day, 5 d/week, 4 weeks, 40 sessions	At baseline and post training	10 m walkway, No info on trials	Fastest velocity	In-shoe plantar pressure measurement system, Parotec system	Assistive device allowed
Lee 2019	14 (7/7)	62 ± 8	49 ± 9	2*	12 (7/5)	62 ± 6	50 ± 10	*	Ability to walk without personal assistance (FAC 3–4)	Gait Enhancing and Motivating System (GEMS)	o-RAGT + t-RAGT	Treadmill gait training + over ground gait training	45 min/d, 3d/week, 4 weeks, 10 sessions	At baseline and post training	8 m walkway, 5 trials	Self-selected velocity	3D motion capture system with 6 infrared cameras	Not reported
Lewek 2009	10 (4/6)	52 ± 12	45 ± 56	1	9 (4/5)	53 ± 6	65 ± 68	5 (+ 1)	Ability to walk at least 10 m over ground without physical assistance and at a self-selected gait speed of 0.8 m/s	Lokomat	t-RAGT with BWS	Treadmill gait training with BWS	60 min, 3d/week, 4 weeks, 12 sessions	At baseline and post training	10 m walkway, over ground, > 5 trials	Self-selected velocity	8-camera motion picture system	Orthoses allowed
Ogino 2020	9 (6/3)	66 ± 10	96 ± 60	1	11 (9/2)	65 ± 8	84 ± 48	0	Ability to walk without physical assistance using assistive devices and braces as needed	Gait Exer-cise Assist Robot (GEAR)	t-RAGT with BWS + limb range motion exercise	Treadmill gait training	60 min/day, 5 d/week, 4 weeks	At baseline and post training, at a 1 month and 3 months follow up	Walk on treadmill, no further info	Self-selected velocity	KinemaTracer, 3D motion analysis system	Handrail or brace allowed
Srivastava 2016	6 (4/2)	62 ± 12	54 ± 53	0	6 (5/1)	59 ± 8	15 ± 10	0	No info	Active Leg Exoskeleton, ALEXII	t-RAGT + FES, no BWS	Treadmill gait training with BWS	40 min, 5 d/every other week, 15 sessions	At baseline and post training	Over ground, No further info	Self-selected velocity	Qualisys 8-camera motion capture system	Not reported
Tanaka 2019	21 (13/8)	65 ± 12	104 ± 28	3	20 (14/6)	62 ± 9	93 ± 36	2	Ability o walk independently or with minimal assistance, and ability to complete gait training sessions lasting 10 min or more	Stride Manage-ment Assistance, SMA	o-RAGT	Conventional gait training	60–120 min/day, 10 sessions	At baseline and post training	8,4 m walkway, over ground, > 2 trials	Fastest velocity	WalkWay MW-1000	Assistive device allowed
Westlake 2009	8 (6/2)	59 ± 17	44 ± 27	0	8 (7/1)	55 ± 14	37 ± 20	0	At least unlimited household ambulators (e.g. > 0.3 m/s)	Lokomat	t-RAGT with BWS	Treadmill gait training with BWS	max 60 min, 3 d/week, 4 weeks, 12 sessions	At baseline and post training	5,3 m walkway, ≥ 3 trials	Self-selected and fastest velocity	GAITRite	Assitive device allowed

*SD* standard deviation, *RAGT* robotic-assisted gait training, *t-RAGT* treadmill-based robotic-assisted gait training, *o-RAGT* overground robotic-assisted gait training, *BWS* body weight support, *FES* functional electric stimulation

The duration of the study interventions ranged from 10 days to 10 weeks, yielding 10–40 sessions, given with a frequency of two to five times per week. Duration ranged from 30 to 105 min per day. Comparator and intervention groups were offered training with similar duration and frequency. Details of the received training (e.g., intensity) were however vague or inadequately reported in many studies. This was specifically true for the training received by the comparator group, where the interpretation of “conventional gait training” or “traditional gait training” may have differed.

The dropout rate during the intervention period ranged from 0 to 37%. In six studies, all included participants completed the training throughout the whole intervention period [34, 38, 40, 42, 46, 48]. Reasons for withdrawal included fear of falling, skin lesions, leg pain due to training, problems with an orthosis, pitting oedema, injury related to training, or self-reported exercise intolerance [37, 43–45, 47]. Dropout was also due to travel limitations and medical or personal reasons that were reported to be unrelated to training [37, 41, 43–45, 47].

All studies performed a baseline and post-intervention assessment within a couple of weeks after the participants completed the training period. In addition, one study [34] included a 2-week follow-up evaluation, while four studies [41, 44, 46, 47] performed follow-up evaluations 1–6 months post-intervention to investigate the long-term effects of RAGT. To collect biomechanical data, gait analysis was performed on a gait mat (GAITRite, Walkway or GaitMat) [34, 38, 40, 44–47], with a 3D motion capture system [38, 39, 41, 42], an accelerometer [48], or an in-shoe plantar pressure measurement system [43]. Of the 13 included studies, biomechanical measures were presented as primary outcomes in nine [37–41, 45–47] and as secondary outcomes in four [34, 42–44].

### Risk of bias assessment

**Fig. 2:**
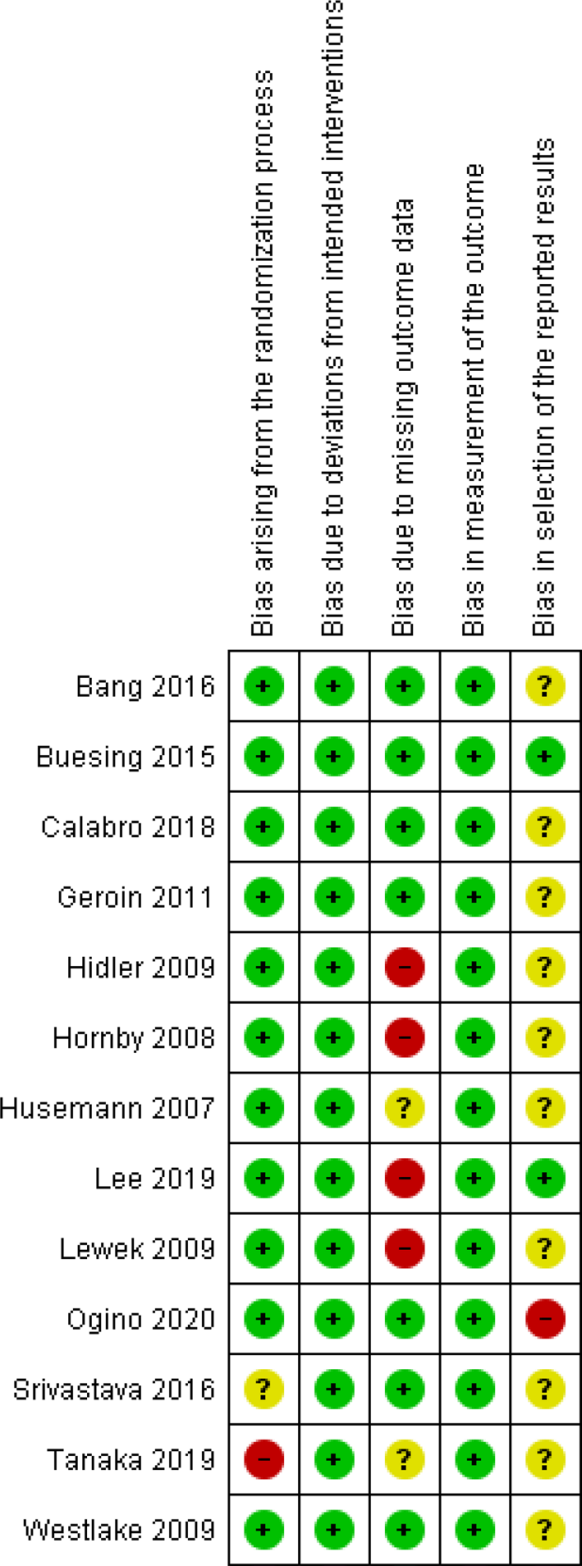
Risk of bias summary: review authors’ assessment of each risk of bias item for every included study (generated with the Review Manager Web, The Cochrane Collaboration, 2019, available at revman.cochrane.org)

**Fig. 3:**
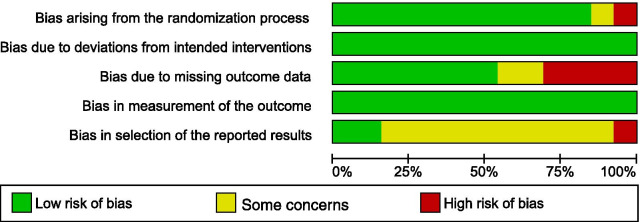
Risk of bias graph (generated with the Review Manager Web, The Cochrane Collaboration, 2019, available at revman.cochrane.org)

A summary of the risk of biases is reported in detail for each included study in Figs. 2 and 3 (generated with Review Manager: Version 5, Copenhagen: The Nordic Cochrane Centre). Risk of bias arising from the randomization process revealed concerns for two studies, either due to a lack of relevant information [42] or that randomization was based on the hospital record numbers of the participants [45] (Fig. 3). Risk of bias due to missing outcome data gave rise to concerns in several studies. Reasons for withdrawal were not reported in one study [39], and when reported they were likely to be related to certain consequences of the training, e.g., fear of falling, skin lesions, leg pain due to training, pitting oedema or self-reported exercise intolerance [37, 43–45, 47]. In all of these studies except for one [43], the dropouts were excluded from the analysis. Selective reporting of results raised some concerns in most of the included studies due to the absence of study protocols or pre-specified analysis plans. Only four studies reported trial registrations [39, 41, 46, 48]. The analysis plan in one of the registered protocols did not conform to the analyses performed in the study [48], and one study [41] chose to report only within-group analyses.

### Effect on temporal and spatial parameters

**Fig. 4:**
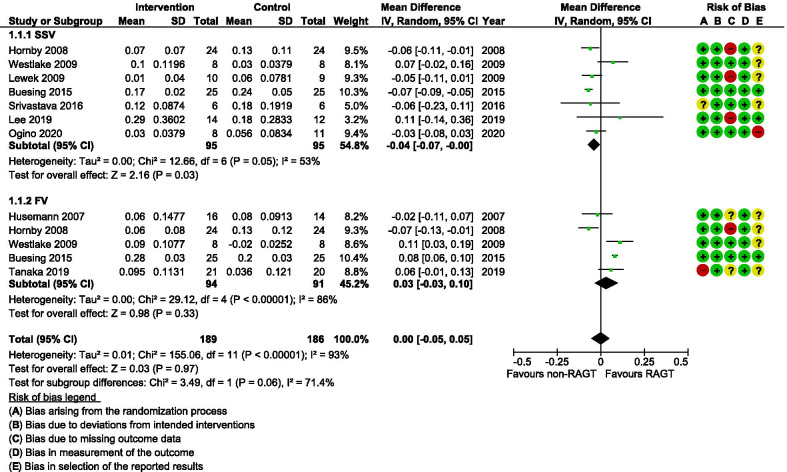
A forest plot (generated with the Review Manager Web, The Cochrane Collaboration, 2019, available at revman.cochrane.org) summarizing a pooled effect estimate on change in *gait speed (m/s)*, following robotic-assisted gait training (RAGT) compared with non-robotic gait training (non-RAGT), during walking at a self-selected velocity (SSV) and the fastest velocity possible (FV). *CI* confidence interval; *df* degrees of freedom; *SD* standard deviation

**Fig. 5:**
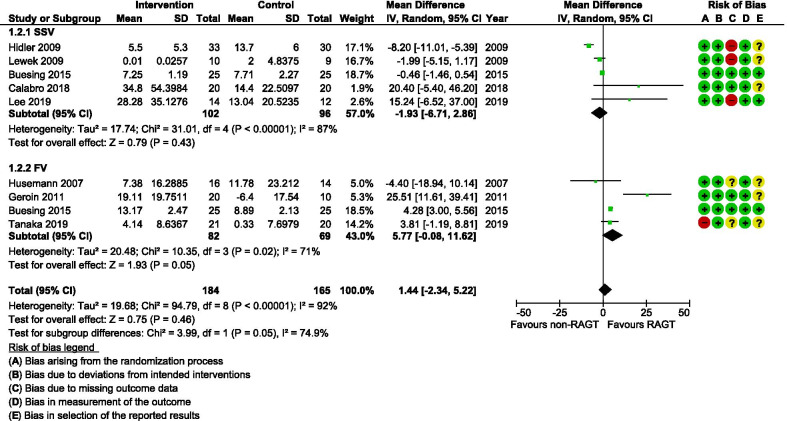
A forest plot (generated with the Review Manager Web, The Cochrane Collaboration, 2019, available at revman.cochrane.org) summarizing a pooled effect estimate on change in *cadence (steps/min)*, following robotic-assisted gait training (RAGT) compared with non-robotic gait training (non-RAGT), during walking at a self-selected velocity (SSV) and the fastest velocity possible (FV). *CI* confidence interval; *df* degrees of freedom; *SD* standard deviation

Although all included studies analysed at least one temporal gait parameter, the most reported were gait speed (n = 10) and cadence (n = 8). The certainty of evidence (GRADE evaluation) was found to be very low for both these parameters because there were some concerns for several studies with regard to the risk of bias (Table 2), indirectness of evidence owing to clinical variation regarding intervention parameters and gait analysis settings (see Table 1), and imprecise findings with insignificant differences between small population groups (6–25 participants/group). The meta-analysis of gait speed (Fig. 4) indicated no overall significant differences between the intervention and comparator groups after the training period (mean difference [MD] 0.00 m/s; 95% confidence interval [CI]   − 0.05, 0.05; I^2^ = 93%). The meta-analysis of cadence similarly did not reveal any significant differences between groups (MD 1.44 steps/min; 95% CI − 2.34, 5.22; I^2^ = 92%) (Fig. 5). Bang et al. [38] did not report gait velocity during assessment (SSV or FV) and this study was therefore excluded from the subgroup meta-analyses. This study found significantly larger improvements for both gait speed (MD 2.14 m/s; 95% CI 0.93, 3.36) and cadence (MD 1.48 steps/min; 95% CI 0.41, 2.55) favouring the RAGT group. When these results were included in the meta-analyses, they did however not influence the overall results.

**Table 2:** A summary of GRADE domains and overall certainty of evidence for each outcome of interest

Outcome	Certainty assessment							No of participants		Effect	Certainty
	№ of studies	Study design	Risk of bias	Inconsistency	Indirectness	Imprecision	Other considerations	RAGT	non-RAGT	Absolute (95% CI)	
Gait speed	8	Randomised trials	Serious^a,b,c,d^	Not serious	Serious^e^	Serious^f^	None	181	175	MD 0 m/s (0.05 lower to 0.05 higher)	⨁◯◯◯ Very low
Cadence	7	Randomised trials	Serious^a,b,c,d^	Not serious	Serious^e^	Serious^f^	None	168	151	MD 1.44 steps/min higher (2.34 lower to 5.22 higher)	⨁◯◯◯ Very low
Other temporal outcomes	6	Randomised trials	Serious^a,b,c^	Serious^g^	Serious^e^	Serious^f^	None	104	112	see comment^	⨁◯◯◯ Very low
Step length	3	Randomised trials	Serious^a,b,c,d^	Not serious	Serious^e^	Serious^f^	None	88	87	MD 1.22 cm higher (0.1 lower to 2.54 higher)	⨁◯◯◯ Very low
Stride length	5	Randomised trials	Serious^a,b,c^	Not serious	Serious^e^	Serious^f^	None	82	79	MD 2.86 cm higher* (0.46 higher to 5.25 higher)	⨁◯◯◯ Very low
Temporal symmetry	4	Randomised trials	Serious^a,b,c^	Not serious	Serious^e^	Serious^f^	None	76	92	MD 0.09 ratio higher* (0.04 higher to 0.15 higher)	⨁◯◯◯ Very low
Spatial symmetry	5	Randomised trials	Serious^a,b,c,d^	Not serious	Serious^e^	Serious^f^	None	149	146	MD 0.01 ratio lower (0.06 lower to 0.04 higher)	⨁◯◯◯ Very low
Kinematics	3	Randomised trials	Serious^a,b,c,d^	Not serious	Not serious	Serious^f^	None	16	15	See comment^	⨁⨁◯◯ Low

Table generated with the GRADEpro Guideline Development Tool. McMaster University, 2015 [developed by Evidence Prime, Inc.]

*RAGT* robotic-assisted gait training, *non-RAGT* non-robotic gait training, *CI* confidence interval, *MD* mean difference

*Indicates significant differences (*p* < 0.05) between groups favouring RAGT

^Statistical pooling was not possible for these variables and the findings are therefore presented in a narrative form in the text

^a^Including at least one study with unclear handling of missing data

^b^Including at least one study with risk for selective outcome reporting

^c^Including one or several studies with overall unclear risk of bias

^d^Including study with high or unclear risk of bias arising from the randomization process

^e^Heterogeneity in intervention settings and gait analysis

^f^Small total population size (< 400)

^g^Downgraded by 1 due to inconsistency in findings across studies

In the subgroup analyses of gait speed and cadence (see Additional files 2 and 3), where the studies employing o-RAGT and those using t-RAGT were differentiated, we did not find any between-group differences. Differences between groups were neither identified in subgroup analyses that differentiated between earlier (2007–2014) and later published studies (2015–2020) with regards to gait speed and cadence (see Additional files 4 and 5).

Other temporal parameters assessed in the included studies were gait cycle/stride duration [43, 48], step time [46], stance time/percentage of GC (single [43, 47] and/or double limb support [38, 41, 43]), and swing time/percentage of GC [41, 46] (Table 3). For the same reasons as for the outcomes used in the meta-analysis, the GRADE assessment indicated very low certainty of evidence for the gathered temporal parameters (Table 2). However, in nearly all studies, no significant difference in temporal parameters between groups was observed and both groups improved to an equal extent. A significantly higher increase in the single-limb stance period favouring the comparator group during FV was reported by Hornby et al. [47] (MD − 3.0%; 95% CI − 6.96, 0.96) (Table 3). In contrast, Bang et al. [38] observed a decreased double limb support time (MD − 1.46%; 95% CI − 2.32, − 0.6) favouring the RAGT group during walking in SSV. Although Calabró et al. [48] reported a significantly higher effect for gait cycle duration in the RAGT group, our calculations (based on measurements of distances in the pdf-file and calculations using RevMan) did not reveal any significant difference between the groups (MD − 0.08 (ratio); 95% CI − 0.19, 0.03) (cf. Tables 3). Finally, the study by Ogino et al. [41] that did not report results of between-group analyses was excluded in the narrative synthesis.

**Table 3:** Evaluated temporal gait parameters and reported results of the included studies

Type of outcome	Velocity during assessment	Parameter	Bang 2016	Buesing 2015	Calabró 2018	Geroin 2011	Hidler 2009	Hornby 2008	Husemann 2007	Ogino 2020	Lee 2019	Lewek 2009	Srivastava 2016	Tanaka 2019	Westlake 2009
Temporal parameters	*SSV*	Gait speed	**R**^ (p = .00)	X				**C** (p = .03)		NR	**R** (p = .00)	C	X		X
		Cadence	**R**^ (p = .00)	X	**R** (p = .00)		X				**R** (p = .00)	X			
		Stride/Gait cycle duration			**R** (p = .00)										
		Step time		X											
		Stance time		X						NR					
		Swing time		X						NR					
		Single limb stance time						X							
		Double support time	**R**^ (p = .01)	X						NR					
	*FV*	Gait speed		X				**C** (p = .02)	X					**R** (p = .01)	X
		Cadence		X		**R** (p = .00)			X					X	
		Stride/Gait cycle duration							X						
		Step time		X											
		Stance time		X					X						
		Swing time		X											
		Single limb stance time						**C** (p = .00)	X						
		Double support time		X											

X = No differences between groups; **R** = Significant difference between groups favouring robotic-assisted gait training; **C** = Significant difference between groups favouring the comparator  group (non-robotic gait training); NR = No reporting of between-group analyses; SSV = Self Selected Velocity; FV = Fastest Velocity

^Velocity not specified in the study

**Table 4:** Evaluated spatial, symmetry and kinematic gait parameters and results of the included studies

Type of outcome	Velocity during assessment	Parameter	Bang 2016	Buesing 2015	Calabró 2018	Geroin 2011	Hidler 2009	Hornby 2008	Husemann 2007	Ogino 2020	Lee 2019	Lewek 2009	Srivastava 2016	Tanaka 2019	Westlake 2009
Spatial parameters	*SSV*	Step length (impaired side)	**R**^/^^ (p = .03)	**R** (p = .05)						NR					
		Step length (non-impaired side)		X						NR					
		Stride length		X						NR	**R** (p = .01)	X			
	*FV*	Step length (impaired side)		X										X	
		Step length (non-impaired side)		X										X	
		Stride length		X											
Symmetry evaluations	*SSV*	Temporal symmetry		X	**R** (p = .00)						**R** (p = .03)				
		Spatial symmetry		X				X			**R** (p = .04)				X
	*FV*	Temporal symmetry		X		**R** (p = .02)									
		Spatial symmetry		**C** (p = .05)				X						X	X
Kinematic parameters	*SV*	Hip, knee and ankle joint angles											X		
		Relation between ankle and hip joint position (circumduction)										X			
		Abnormal gait pattern ~								NR					

X = No differences between groups; **R** = Significant difference between groups favouring robotic-assisted gait training; **C** = Significant difference between groups favouring the comparator group (non-robotic gait training); NR = No reporting of between-group analyses; SSV = Self Selected Velocity; FV = Fastest Velocity

^Velocity not specified in the study

^^No information on which side has been analysed (affected or non-affected)

^~^Investigated gait patterns were: circumduction, retropulsion of the hip, excessive hip external rotation, knee extensor thrust, flexed-knee gait, insufficient knee flexion during swing phase, forefoot contact, medial whip, excessive lateral shift of the trunk over the unaffected side

**Fig. 6:**
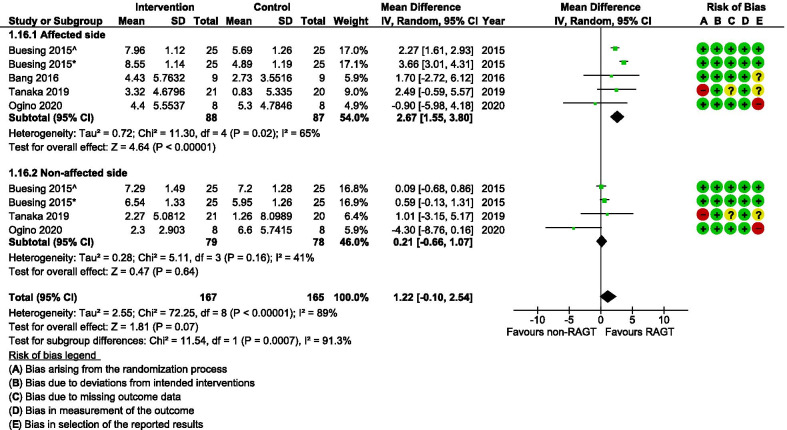
A forest plot (generated with the Review Manager Web, The Cochrane Collaboration, 2019, available at revman.cochrane.org) summarizing a pooled effect estimate on change in *step length (cm)*, following robotic-assisted gait training (RAGT) compared with non-robotic gait training (non-RAGT). *: assessed during walking at a self-selected velocity (SSV); ^: assessed during walking at the fastest velocity possible (FV); *CI* confidence interval; *df* degrees of freedom; *SD* standard deviation

**Fig. 7:**
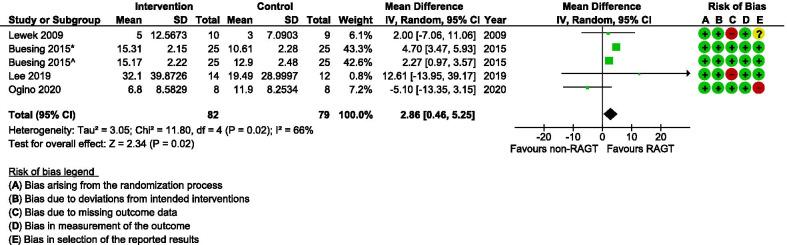
A forest plot (generated with the Review Manager Web, The Cochrane Collaboration, 2019, available at revman.cochrane.org) summarizing a pooled effect estimate on change in *stride length (cm)*, following  robotic-assisted gait training (RAGT) compared with non-robotic gait training (non-RAGT). *: assessed during walking at a self-selected velocity SSV; ^: assessed during walking at the fastest velocity possible FV; *CI* confidence interval; *df* degrees of freedom; *SD* standard deviation

Among spatial parameters, step length increased significantly more on the affected side following RAGT compared with non-robotic gait training (MD 2.67 cm; 95% CI 1.55, 3.80; I^2^ = 65%) (Fig. 6). Such between-group differences were not detectable for step length on the non-affected side, nor for the combined (affected and non-affected side) change. As seen in Fig. 7, stride length increased significantly more in the RAGT group when compared with the comparator group (MD 2.88 cm; 95% CI 0.46, 5.25; I^2^ = 66%). However, the (GRADE) certainty of evidence remained very low for both these parameters (Table 2).

**Fig. 8:**
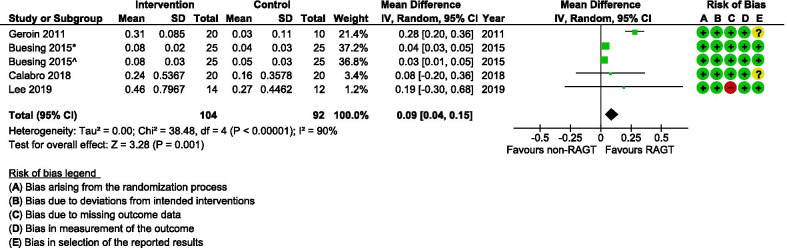
A forest plot (generated with the Review Manager Web, The Cochrane Collaboration, 2019, available at revman.cochrane.org) summarizing a pooled effect estimate on change in *temporal symmetry (ratio)*, following robotic-assisted gait training (RAGT) compared with non-robotic gait training (non-RAGT). *: assessed during walking at a self-selected velocity SSV; ^: assessed during walking at the fastest velocity possible FV; *CI* confidence interval; *df* degrees of freedom; *SD* standard deviation

**Fig. 9:**
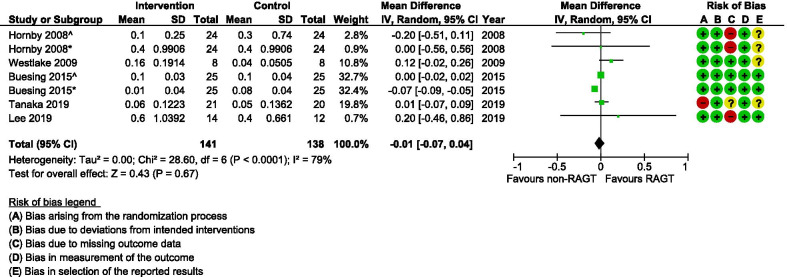
A forest plot (generated with the Review Manager Web, The Cochrane Collaboration, 2019, available at revman.cochrane.org) summarizing a pooled effect estimate on change in *spatial symmetry (ratio)*, following robotic-assisted gait training (RAGT) compared with non-robotic gait training (non-RAGT). *: assessed during walking at a self-selected velocity SSV; ^: assessed during walking at the fastest velocity possible FV; *CI* confidence interval; *df* degrees of freedom; *SD* standard deviation

Seven studies [34, 37, 39, 40, 45, 46, 48] calculated some kind of temporal or spatial symmetry ratio (Table 4) by using a variety of ratio calculations of different spatio-temporal parameters for either or both the paretic and non-paretic limbs. Results from the meta-analysis of temporal symmetry (Fig. 8) revealed very low evidence (Table 2) for a statistically significant improvement in the symmetry ratio in the RAGT groups compared with the non-robotic gait training groups (MD 0.09; 95% CI 0.04, 0.15; I^2^ = 90%). For spatial asymmetry (Fig. 9) on the other hand, no significant differences were observed between groups (MD − 0.01; 95% CI − 0.06, 0.04; I^2^ = 80).

### Effect on kinematic parameters

Only three of the included studies [37, 41, 42] analysed kinematic data, and the overall (GRADE) certainty of evidence was found to be low for the kinematic parameters (Table 4). Lewek et al. [37] detected the consistency of intra-limb hip and knee angular trajectories over repeated gait cycles and the maximum lateral deviation of the heel during the swing phase with respect to the position of the ipsilateral heel during consecutive stance phases (known as circumduction). Srivastava et al. [42] analysed the peak flexion angles during the swing phase. Both studies analysed gait at SSV but none of them found any differences between groups after the intervention period. Ogino et al. [41] used kinematic data to detect index values for abnormal gait patterns following stroke. However, they did not report results from between-group analyses.

### Effect on kinetic parameters

Kinetic variables represent the forces generating the kinematics and spatiotemporal outcomes during walking, and kinetic information should therefore be very useful for understanding and interpreting gait characteristics [49]. However, we did not find any eligible RCTs evaluating kinetic gait data following RAGT.

### Follow-up data

Four studies performed a follow-up test: one after 2 weeks [34], one after 1 month [41], three after 3 months [41, 44, 46] and one after 6 months [47]. The outcome measures investigated during the follow-up period were gait speed, cadence, step time, swing/stance time, step length, temporal and spatial symmetry and gait kinematics (Tables 3 and 4). The 2 week follow-up by Geroin et al. [34], found similar group differences in cadence and temporal symmetry that had been observed during the assessments immediately after the intervention period. According to this, the RAGT group showed a significant improvement in cadence (MD 15.6 steps/min; 95% CI 8.15, 23.11) and temporal symmetry (MD − 0.42 (ratio); 95% CI − 0.5, − 0.34) when compared with the comparator group. No group differences were otherwise detected for any of the investigated outcomes when RAGT was compared with non-robotic gait training during follow-up measurements [44, 46, 47].

## Discussion

Except for synthesised evaluations of gait speed, this is the first study to review and ascertain the level of evidence for RAGT with the quantification of post-stroke gait quality based on biomechanical measures. While employing a pre-registered comprehensive search strategy, based on well-defined eligibility criteria for studies, in nine renowned databases and screening 2857 retrieved citations, only 13 RCTs met the eligibility criteria to address the research question.

Analysis and synthesis of the included studies revealed mixed effects on biomechanical measures assessed after RAGT. Risk of bias assessment raised concerns for several of the studies due to limitations in the randomization process and poor reporting regarding the handling of missing data (See Figs. 2 and 3). Furthermore, reporting bias could be a problem as the plan for analysis was seldom available and only three studies [39, 46, 48] reported a registered study protocol. The variety of outcome measures used in the included studies limited the ability to pool results. However, meta-analyses for gait speed and cadence showed no effect of RAGT that exceeded the effect of non-robotic treatment (Figs. 4 and 5)*.* Owing to the uncertainty of the evidence, specifically concerning the risk of bias, small population sizes, and heterogeneity and inconsistency in results, the general quality of evidence for these outcomes was downgraded to very low despite including only randomized controlled trials (RCTs) (Table 2). The low number of eligible RCTs identified for inclusion in this review, in combination with the concerning risk of bias associated with them, rendered a low certainty of evidence for the effects of RAGT on gait speed among persons with stroke compared with non-robotic training. Methodologically robust RCTs are required to elucidate any potential effects of RAGT on biomechanical parameters relating to post-stroke gait.

Evidence for the potential of RAGT, in combination with physiotherapy, to increase the likelihood of regaining independent walking ability post-stroke has previously been reported by Mehrholtz et al. to be moderate [12]. Their findings, as well as the findings of yet another review [14], were in line with ours and showed that gait speed (assessed either with clinical or instrumented methods) improved to an equal extent in the RAGT and comparator groups post-stroke.

We found the certainty of evidence for the effect of RAGT on cadence to be very low for the same reasons as mentioned for gait speed (Table 2). The pooled results revealed an equal amount of improvement in cadence in intervention and comparator groups (Fig. 5). Gait speed and cadence on their own do not reflect the specific gait movement pattern during walking and should instead be interpreted in combination with other spatial and/or temporal kinematic parameters [21, 50]. Step length and stride length are considered closely connected to gait speed [74]. However, even though a difference between groups was not detectable with regards to gait speed, our analyses showed that step length (Fig. 6) and stride length (Fig. 7) on the affected side improved more in the RAGT group when compared with the non-robotic training group.

The typical motor impairments following stroke cause an unbalanced walking pattern which is often characterized by asymmetries in temporal parameters (relation between affected and non-affected swing, stance, step and/or stride time) and/or spatial parameters (relation between affected and non-affected limb positions and/or step length) [51, 52]. The consequences of spatial and temporal asymmetry are debated [53], but symmetry ratios seem to be more sensitive measures of recovery compared with absolute values of spatial or temporal parameters [54]. Gait symmetry is further associated with a high energy expenditure [55], a high risk of falls, and an unequal loading of the joints (increasing the risk of joint degeneration and bone density loss in the paretic limb) [56]. Our meta-analysis showed that temporal symmetry improved slightly more in the RAGT groups when compared with the non-robotic gait training groups (Fig. 8). There were conversely no significant differences observed between groups with regard to spatial symmetry after training (Fig. 9). The pattern of spatial symmetry vary greatly, while some individuals exhibit a longer step length on the non-paretic side, and others take longer steps on the paretic side [57, 58]. A step lengthening strategy on the affected side detected for the RAGT group (see Fig. 6) may not necessarily be associated with a similar improvement in spatial symmetry in the same group. The level of asymmetry is suggested to be more relevant than some other parameters (e.g., gait speed and step length) to identify the degree of impairment and compensatory mechanisms used during walking post-stroke [59]. These results should however be interpreted cautiously since the GRADE certainty of evidence was very low due to inconclusive results between studies and since there were only a few studies with relatively small sample sizes that investigated this.

Meta-analyses that differentiated studies from before and after 2015 found no differences between the groups with regards to gait speed and cadence (see Additional files 4 and 5). However, when contrasting the findings of most recent studies with those of earlier research (Tables 3 and 4), the most recent studies (published from 2018 to 2019) reported larger improvements of spatial and temporal parameters in the RAGT groups when compared with the non-robotic training groups [39, 45, 48]. This may reflect how increased knowledge in the area of gait rehabilitation post-stroke, combined with the rapid technological development of advanced robotic devices, may have improved RAGT post-stroke. Several factors are considered imperative to stimulate increased neural activity and reorganization: (1) enhancement of active wearer participation, which includes the possibility of individual adjustments and an adaptable robotic interference [3, 8, 60, 61], (2) sufficient degrees of freedom to minimize motion restrictions in the joints and allow other aspects of gait, such as balance, to be incorporated in training [3, 8, 60, 61], and (3) real-time biofeedback to the wearer [8]. The trend towards more positive results for the biomechanical gait measures in the most recent studies evaluating RAGT post-stroke might be a result of adjustments made based on the factors mentioned above. For instance, Lee et al. (2019) [39] and Calabrò et al. (2018) [48] provided active wearer participation through individual guiding, encouragement, adaptation and progression.

Low certainty of evidence indicated that kinematic measures were not significantly different between the groups after the intervention [37, 42]. Although kinematic measurements could help to discriminate between restoration and compensation strategies that develop post-stroke, only two studies [37, 42] that collected kinematic data after RAGT (Tables 3 and 4) were found. Hence, no certain conclusions can be made regarding the effect of RAGT on kinematic measures of gait post-stroke at this time. It has been advocated that detailed kinematic data should be collected and analysed for quantification of specific gait movement patterns when investigating walking post-stroke [18, 20]. Even so, the need for sophisticated laboratory equipment, competence of assessors, and more time and finances may be some reasons for the lack of studies that have performed these assessments.

The requirements in terms of finance, assessor competence and time are similar for the collection of kinetic and kinematic data. To obtain valid kinetic data representing joint moments and power, the individual also needs to walk independently without walking aids, as naturally as possible, and contact the force platform with only one foot. This may be a challenging task for individuals post-stroke who have severe impairments. We did not find any eligible RCTs that evaluated kinetic variables after RAGT. Spatial and temporal parameters during walking are nevertheless direct consequences of the kinetic parameters affecting the joints [49]. Several studies have shown kinetic deviations in both the affected and unaffected leg in individuals post-stroke [62, 63]. Moments and power bursts are suggested to be reduced in amplitude in persons post-stroke when compared with asymptomatic controls while walking at a self-selected speed [64]. However, the total lack of eligible robust RCTs evaluating kinetic variables after RAGT post-stroke calls for future research in this area.

## Methodological considerations

This review followed a pre-registered protocol in PROSPERO, included relevant RCTs published until the 19th of January 2021, employed the updated and comprehensive Cochrane risk of bias 2 tool (2019), and summarized the current level of evidence for the biomechanical gait parameters of interest using the established GRADE criteria. In general, the RAGT and comparator groups were comparable based on the amount of therapy (duration and frequency) provided, but information on the intensity (here defined as the amount of work per unit time [i.e., the rate of work or power]) of training and individual adjustments was inadequate or unavailable*.* The importance of specifying the intensity of practice has been emphasized [65]. The intensity is nonetheless applied and specified inconsistently in most exercise training protocols. This is also the case for the studies included in this review, and this may account for some of the differences in the conclusions regarding the lack of superiority of one rehabilitation method compared with the other. Clinical heterogeneity between the included studies was also considerable due to differences in the population of interest (stroke severity level and phase post-stroke, etc.) and intervention settings (the robotic device used, feedback delivered, and content, duration, intensity and frequency of the training in both the RAGT as well as the comparator groups).

The chosen biomechanical outcome measures (different spatial and/or temporal parameters) and the settings for gathering gait data varied (various systems for gait analysis, allowance of walking aids during assessments, variations in walking distances, etc.). This variation together with different definitions and/or calculations in data analysis limit the possibility of generalising the results. We also did not include studies using only electromyography (EMG) in our review because motor intent identification using EMG may have significant limitations in individuals post-stroke due to severe motor impairment, profound muscle fatigue, or abnormally coactivated muscles [66, 67]. This is further corroborated in an up-to-date compilation of evidence in this area provided by Lennon et al. (2020) [68]. Finally, this review excluded papers in languages other than English.

It has been suggested that the effect of RAGT depends on factors such as time after stroke and impairment severity [12]. Indeed, RAGT combined with physiotherapy has been suggested to be especially efficient in improving the function and mobility of the lower limbs in non-ambulatory patients in their subacute phase post-stroke [11, 13]. It has also been hypothesized that gait function and movement pattern is less likely to change in a chronic phase post-stroke. Interventions for improving motor performance, initiated in an early stage post-stroke, are assumed to closely interact with the dynamic phases of neural remodelling to promote better reorganization [69]. Hence, the possibility to influence the gait movement pattern post-stroke would supposedly decrease with the course of time post-stroke. Only three of the thirteen studies included in this review had a population in a subacute phase post-stroke, whereas the others addressed populations in a chronic phase. Since the assessment of gait biomechanics requires walking function (with or without aids or personal support), none of the studies included non-ambulatory persons. It was thus not possible to draw any conclusions regarding the impact of RAGT in relation to the different phases post-stroke (subacute/chronic) or different severity levels of impairments.

Another topic of interest when evaluating the effects of RAGT is the possible difference between RAGT employed on a treadmill and the one performed overground. Overground RAGT is hypothesized to provide greater motor control stimulation, multisensory plasticity and required effort when compared with RAGT performed on a treadmill [5]. Hence, we performed subgroup-analyses based on the employed type of RAGT for the outcomes of gait-speed and cadence but found no significant differences between the groups (Fig. 6).

## Future challenges and recommendations

Robotics in gait training post-stroke requires an evaluation from several perspectives in order to identify responders and non-responders to RAGT (e.g., the impact of RAGT in relation to the different phases post-stroke and different severity levels of impairments) and map the strengths and weaknesses to support and guide future technical development. Highly intensive, task-specific and repetitive gait training post-stroke, such as RAGT, is assumed to stimulate restoration of motor skills and, consequently, normalize the gait movement pattern through neuroplasticity [70–72]. Yet this has not been thoroughly investigated and the mechanisms underlying functional gains achieved through RAGT in individuals post-stroke are still poorly understood. This review highlights the need to combine the measures of task accomplishment with objective assessments of gait movement patterns and gait quality after RAGT.

A consensus is unfortunately lacking as to which biomechanical gait measures to use when investigating motor coordination and the quality of movement patterns during walking [50, 73, 74]. Standardised guidelines for assessing and reporting gait variables should be developed to support researchers and enable pooling of results to facilitate the evaluation of the effects of and further development of gait-assisting robots used in post-stroke rehabilitation. These guidelines should include assessments of several (spatial, temporal and kinetic) aspects of gait [15, 50], and consider bilateral motor coordination [50] as well as the engagement of the trunk [75] and displacement of centre of mass [76]. Inter-limb coordination, such as symmetry in spatio-temporal parameters, including kinematic measures of movement endpoint, whole trajectories, and joint angles, as well as in kinetic parameters, are all important outcomes reflecting the quality of gait post-stroke [76] and bilateral motor coordination [50].

The challenge of bioengineers is to match the most recent neurological findings with the features of the robots developed for gait training post-stroke [3]. These robots should not only simulate physiological patterns but also favour the determinants of a qualitative gait recovery. To stimulate the recovery of a close-to-normal gait movement pattern, the robots should enable variability in lower limb kinematics through sufficient degrees of freedom in all three planes of motion [60]. In addition, they should be flexible and individually adjustable, and they need to encourage active participation from the wearer. The combination of individual support and progression, realtime feedback and guidance, and motor tasks that challenge balance control and coordination, serves for multisensory stimulation that has been suggested to be beneficial for neural reorganization. The optimally developed robot should have the ability to generate a bottom-up and top-down complex and controlled multisensory stimulation aiming to modify the plasticity of neural connections through the experience of moving [5].

## Conclusion

This systematic review revealed a substantial knowledge gap underpinning the effects of RAGT post-stroke when compared with non-robotic gait training. Only thirteen eligible randomised controlled trials were identified which evaluated the effects of RAGT post-stroke on objective biomechanical outcome measures. Our findings demonstrated a very low certainty in current evidence for employing RAGT instead of non-robotic gait training to improve gait ability post-stroke. Standardised guidelines for biomechanical quantification of gait should be developed to support researchers in the evaluation of gait-assisting robots used in post-stroke rehabilitation. Well-designed, high-quality clinical trials that complement clinical data with objective, quantitative gait data post-stroke will provide more detailed information on the potential effects of robotic gait training in general, as well as the influence on gait movement pattern in particular. In the long term, this could contribute to the development of RAGT that, either on its own or as an addition to other treatments, can better target true recovery and decrease the impact of compensatory gait patterns post-stroke.

## Supplementary Information

**Additional file 1**: A summary of search terms and results from different databases (Web of Science, Scopus, PubMed, the Cochrane Central Register of Controlled Trials (CENTRAL), Academic Search Premier, Cumulative Index to Nursing and Allied Health Literature [CINAHL], Allied and Complementary Medicine [AMED], ProQuest (Sports Medicine & Education Index) and Sports Discus)

**Additional file 2**: A forest plot (generated with the Review Manager Web, The Cochrane Collaboration, 2019, available at revman.cochrane.org) summarizing a pooled effect estimate on change in gait speed (m/s), following robotic-assisted gait training (RAGT) compared with non-robotic gait training (non-RAGT). Subgroup analyses based on the type of gait robots used: treadmill robotic-assisted gait training (t-RAGT) or overground robotic-assisted gait training (o-RAGT). *: assessed during walking at a self-selected velocity SSV; ^: assessed during walking at the fastest velocity possible FV; CI: confidence interval; df: degrees of freedom; SD: standard deviation

**Additional file 3**: A forest plot (generated with the Review Manager Web, The Cochrane Collaboration, 2019, available at revman.cochrane.org) summarizing a pooled effect estimate on change in cadence (steps/min), following robotic assisted gait training (RAGT) compared with non-robotic gait training (non-RAGT). Subgroup analyses based on the type of gait robots used: treadmill robotic-assisted gait training (t-RAGT) or overground robotic-assisted gait training (o-RAGT). *: assessed during walking at a self-selected velocity SSV; ^: assessed during walking at the fastest velocity possible FV; CI: confidence interval; df: degrees of freedom; SD: standard deviation

**Additional file 4**: A forest plot (generated with the Review Manager Web, The Cochrane Collaboration, 2019, available at revman.cochrane.org) summarizing a pooled effect estimate on change in gait speed (m/s), following robotic assisted gait training (RAGT) compared with non-robotic gait training (non-RAGT). Subgroup analyses based on the year of publication: late studies published 2015–2020 and early studies 2007–2014. *: assessed during walking at a self-selected velocity SSV; ^: assessed during walking at the fastest velocity possible FV; CI: confidence interval; df: degrees of freedom; SD: standard deviation

**Additional file 5**: A forest plot (generated with the Review Manager Web, The Cochrane Collaboration, 2019, available at revman.cochrane.org) summarizing a pooled effect estimate on change in cadence (steps/min), following robotic assisted gait training (RAGT) compared with non-robotic gait training (non-RAGT). Subgroup analyses based on the year of publication: late studies published 2015-2020 and early studies 2007-2014. *: assessed during walking at a self-selected velocity SSV; ^: assessed during walking at the fastest velocity possible FV; CI: confidence interval; df: degrees of freedom; SD: standard deviation

## Data Availability

Full search strategy available in Appendix. The datasets analysed during the current study are available from the corresponding author on reasonable request.

## Abbreviations

CI: Confidence interval
FES: Functional electrical stimulation
FV: Fastest possible velocity
GC: Gait cycle
GRADE: Grading of assessment, development and evaluation
ICF: International classification of functioning, disability and health
MD: Mean difference
o-RAGT: Over ground robotic assisted gait training
PRISMA: Preferred reporting items for systematic review and meta-analysis
RAGT: Robotic assisted gait training
RCT: Randomized clinical trial
SSV: Self-selected velocity
t-RAGT: Treadmill-based robotic assisted gait training
3D: 3-Dimensional
2D: 2-Dimensional
