# Supplementary material for: Effect of robotic-assisted gait training on objective biomechanical measures of gait in persons post-stroke: a systematic review and meta-analysis
Source: J Neuroeng Rehabil. 2021 Apr 16;18:64. doi: 10.1186/s12984-021-00857-9 (PMC8052671; doi:10.1186/s12984-021-00857-9)
Supplement: Supplementary file 1 — Additional file 1: A summary of search terms and results from different databases (Web of Science, Scopus, PubMed, the Cochrane Central Register of Controlled Trials (CENTRAL), Academic Search Premier, Cumulative Index to Nursing and Allied Health Literature [CINAHL], Allied and Complementary Medicine [AMED], ProQuest (Sports Medicine & Education Index) and Sports Discus) [file 12984_2021_857_MOESM1_ESM.docx]

| **Database** | **Search terms** | **Records** |
| --- | --- | --- |
| Web of Science | TOPIC: (Exoskelet* OR End-effector* OR Robot* OR Gait-trainer* OR Loko* OR G-EO system) AND TOPIC: (Locomot* OR walk* OR ambulation OR gait OR postural balance) AND TOPIC: (Kinematic* OR kinetic* OR Motion analys* OR Gait analys* OR 2-D OR 3-D OR Three dimensional OR Two dimensional OR Force OR Instrument* OR Joint position* OR Movement analys* OR spati* OR temporal OR symmetr* OR asymmetr* OR dissymmetry* OR Biomechanical phenomena) AND TOPIC: (Post-stroke OR Brain infarction OR Hemipares* OR stroke OR Paresis OR hemiplegia OR Cerebrovascular disorders) Refined by: LANGUAGES: ( ENGLISH ) | 807 |
| Scopus | TITLE-ABS-KEY ( locomot* OR walk* OR ambulation OR gait OR "postural balance" ) AND ( TITLE-ABS-KEY ( post-stroke OR "Brain infarction" OR hemipares* OR stroke OR paresis OR hemiplegia OR "Cerebrovascular disorders" ) ) AND ( TITLE-ABS-KEY ( exoskelet* OR end-effector* OR robot* OR "Gait-trainer*" OR loko* OR "G-EO system" ) ) AND ( TITLE-ABS-KEY ( kinematic* OR kinetic* OR "Motion analys*" OR "Gait analys*" OR 2-d OR 3-d OR "Three dimensional" OR "Two dimensional" OR force OR instrument* OR "Joint position*" OR "Movement analys*" OR spati* OR temporal OR symmetr* OR asymmetr* OR dissymmetry* OR "Biomechanical phenomena" ) ) AND ( LIMIT-TO ( LANGUAGE , "English" ) | 717 |
| PubMed | (((((((Exoskelet*[Title/Abstract] OR End-effector*[Title/Abstract] OR Robot*[Title/Abstract] OR Gait-trainer*[Title/Abstract] OR Loko*[Title/Abstract] OR G-EO system[Title/Abstract])) OR Exoskeleton device[MeSH Terms]) OR Robotics[MeSH Terms])) AND ((((Locomot*[Title/Abstract] OR walk*[Title/Abstract] OR ambulation[Title/Abstract] OR gait[Title/Abstract] OR movement[Title/Abstract] OR postural balance[Title/Abstract])) OR postural balance[MeSH Terms]) OR walking[MeSH Terms])) AND ((((Kinematic*[Title/Abstract] OR kinetic*[Title/Abstract] OR Motion analys*[Title/Abstract] OR Gait analys*[Title/Abstract] OR 2-D[Title/Abstract] OR 3-D[Title/Abstract] OR Three dimensional[Title/Abstract] OR Two dimensional[Title/Abstract] OR Force[Title/Abstract] OR Instrument*[Title/Abstract] OR Joint position*[Title/Abstract] OR Movement analys*[Title/Abstract] OR spati*[Title/Abstract] OR temporal[Title/Abstract] OR symmetr*[Title/Abstract] OR asymmetr*[Title/Abstract] OR dissymmetry*[Title/Abstract] OR Biomechanical phenomena[Title/Abstract])) OR Biomechanical phenomena[MeSH Terms]) OR gait analysis[MeSH Terms])) AND (((((Post-stroke[Title/Abstract] OR Brain infarction[Title/Abstract] OR Hemipares*[Title/Abstract] OR stroke[Title/Abstract] OR Paresis[Title/Abstract] OR hemiplegia[Title/Abstract] OR Cerebrovascular disorders[Title/Abstract])) OR Paresis[MeSH Terms]) OR Cerebrovascular disorders[MeSH Terms]) OR hemiplegia[MeSH Terms]) AND (English[lang]) | 563 |
| Cochrane | Exoskeleton OR End-effector OR Robot* OR Gait-trainer in Title Abstract Keyword AND Locomotion OR walk* OR ambulation OR gait OR movement OR postural balance in Title Abstract Keyword AND Kinematic OR Kinetic OR Motion analysis OR Gait analysis OR 2D OR 3D OR Three dimensional OR Two dimensional OR Force OR Instrument* OR Joint position OR Movement analysis OR Biomechanical phenomena in Title Abstract Keyword AND Post-stroke OR Brain infarction OR hemiplegia OR Hemiparesis OR Cerebrovascular disorder OR stroke OR Paresis OR Cerebellar diseases in Title Abstract Keyword | 351 trials |
| Academic Search Premier | AB ( Exoskelet* OR End-effector* OR Robot* OR Gait-trainer* OR Loko* OR G-EO system ) AND AB ( Locomot* OR walk* OR ambulation OR gait OR postural balance ) AND AB ( Kinematic* OR kinetic* OR Motion analys* OR Gait analys* OR 2-D OR 3-D OR Three dimensional OR Two dimensional OR Force OR Instrument* OR Joint position* OR Movement analys* OR spati* OR temporal OR symmetr* OR asymmetr* OR dissymmetry* OR Biomechanical phenomena ) AND AB ( Post-stroke OR Brain infarction OR Hemipares* OR stroke OR Paresis OR hemiplegia OR Cerebrovascular disorders ) | 161 |
| CINAHL | AB ( Exoskelet* OR End-effector* OR Robot* OR Gait-trainer* OR Loko* OR G-EO system ) AND AB ( Locomot* OR walk* OR ambulation OR gait OR postural balance ) AND AB ( Kinematic* OR kinetic* OR Motion analys* OR Gait analys* OR 2-D OR 3-D OR Three dimensional OR Two dimensional OR Force OR Instrument* OR Joint position* OR Movement analys* OR spati* OR temporal OR symmetr* OR asymmetr* OR dissymmetry* OR Biomechanical phenomena ) AND AB ( Post-stroke OR Brain infarction OR Hemipares* OR stroke OR Paresis OR hemiplegia OR Cerebrovascular disorders ) | 128 |
| Sports Medicine & Education Index | ab(Exoskelet* OR End-effector* OR Robot* OR Gait-trainer* OR Loko* OR G-EO system ) AND ab(Locomot* OR walk* OR ambulation OR gait OR postural balance ) AND ab(Kinematic* OR kinetic* OR Motion analys* OR Gait analys* OR 2-D OR 3-D OR Three dimensional OR Two dimensional OR Force OR Instrument* OR Joint position* OR Movement analys* OR spati* OR temporal OR symmetr* OR asymmetr* OR dissymmetry* OR Biomechanical phenomena ) AND ab(Post-stroke OR Brain infarction OR Hemipares* OR stroke OR Paresis OR hemiplegia OR Cerebrovascular disorders) | 58 |
| AMED | AB ( Exoskelet* OR End-effector* OR Robot* OR Gait-trainer* OR Loko* OR G-EO system ) AND AB ( Locomot* OR walk* OR ambulation OR gait OR postural balance ) AND AB ( Kinematic* OR kinetic* OR Motion analys* OR Gait analys* OR 2-D OR 3-D OR Three dimensional OR Two dimensional OR Force OR Instrument* OR Joint position* OR Movement analys* OR spati* OR temporal OR symmetr* OR asymmetr* OR dissymmetry* OR Biomechanical phenomena ) AND AB ( Post-stroke OR Brain infarction OR Hemipares* OR stroke OR Paresis OR hemiplegia OR Cerebrovascular disorders ) | 35 |
| SPORT Discus | AB ( Exoskelet* OR End-effector* OR Robot* OR Gait-trainer* OR Loko* OR G-EO system ) AND AB ( Locomot* OR walk* OR ambulation OR gait OR postural balance ) AND AB ( Kinematic* OR kinetic* OR Motion analys* OR Gait analys* OR 2-D OR 3-D OR Three dimensional OR Two dimensional OR Force OR Instrument* OR Joint position* OR Movement analys* OR spati* OR temporal OR symmetr* OR asymmetr* OR dissymmetry* OR Biomechanical phenomena ) AND AB ( Post-stroke OR Brain infarction OR Hemipares* OR stroke OR Paresis OR hemiplegia OR Cerebrovascular disorders ) | 37 |
