# Supplementary figures and images for: Effect of robotic-assisted gait training on objective biomechanical measures of gait in persons post-stroke: a systematic review and meta-analysis
Source: J Neuroeng Rehabil. 2021 Apr 16;18:64. doi: 10.1186/s12984-021-00857-9 (PMC8052671; doi:10.1186/s12984-021-00857-9)

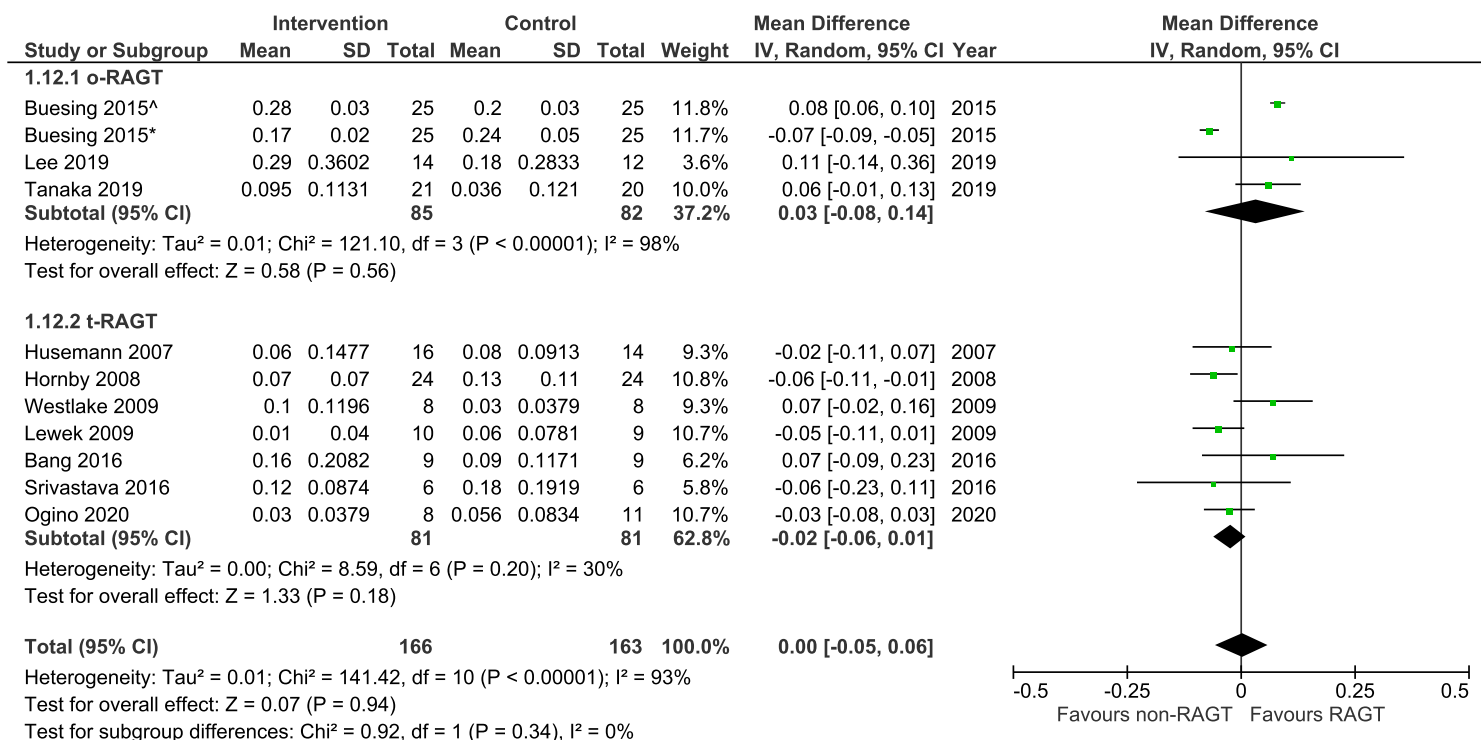

Supplement: Supplementary file 2 — Additional file 2: A forest plot (generated with the Review Manager Web, The Cochrane Collaboration, 2019, available at revman.cochrane.org) summarizing a pooled effect estimate on change in gait speed (m/s), following robotic-assisted gait training (RAGT) compared with non-robotic gait training (non-RAGT). Subgroup analyses based on the type of gait robots used: treadmill robotic-assisted gait training (t-RAGT) or overground robotic-assisted gait training (o-RAGT). *: assessed during walking at a self-selected velocity SSV; ^: assessed during walking at the fastest velocity possible FV; CI: confidence interval; df: degrees of freedom; SD: standard deviation [file 12984_2021_857_MOESM2_ESM.pdf]

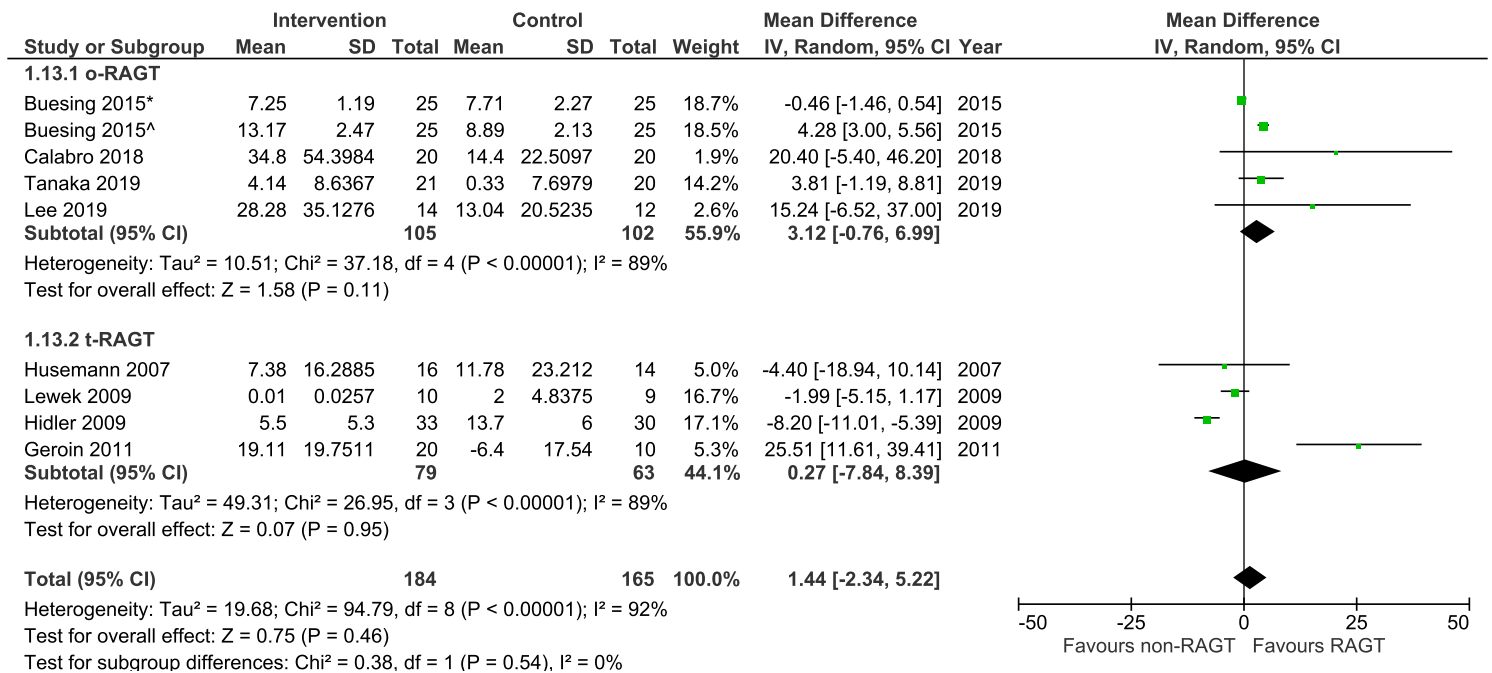

Supplement: Supplementary file 3 — Additional file 3: A forest plot (generated with the Review Manager Web, The Cochrane Collaboration, 2019, available at revman.cochrane.org) summarizing a pooled effect estimate on change in cadence (steps/min), following robotic assisted gait training (RAGT) compared with non-robotic gait training (non-RAGT). Subgroup analyses based on the type of gait robots used: treadmill robotic-assisted gait training (t-RAGT) or overground robotic-assisted gait training (o-RAGT). *: assessed during walking at a self-selected velocity SSV; ^: assessed during walking at the fastest velocity possible FV; CI: confidence interval; df: degrees of freedom; SD: standard deviation [file 12984_2021_857_MOESM3_ESM.pdf]

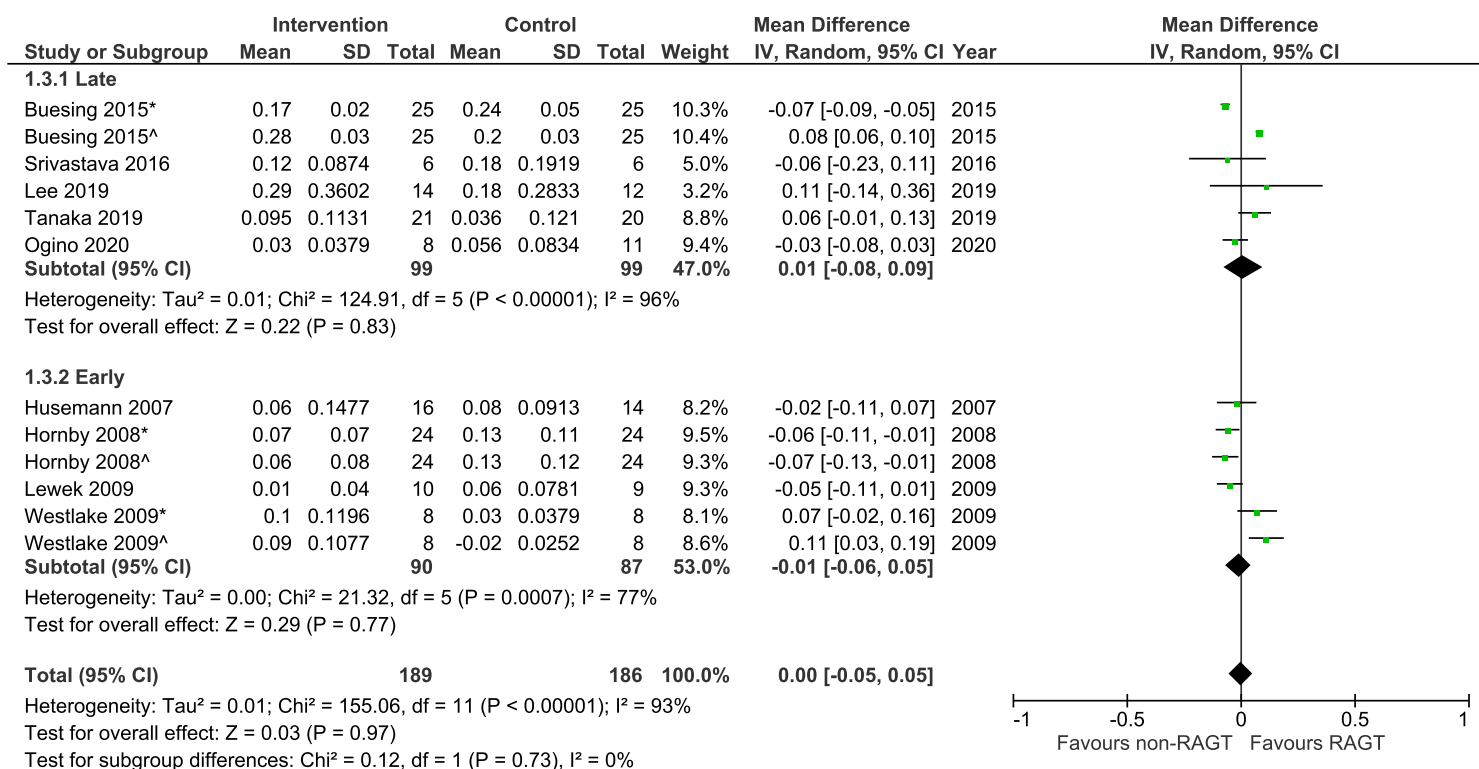

Supplement: Supplementary file 4 — Additional file 4: A forest plot (generated with the Review Manager Web, The Cochrane Collaboration, 2019, available at revman.cochrane.org) summarizing a pooled effect estimate on change in gait speed (m/s), following robotic assisted gait training (RAGT) compared with non-robotic gait training (non-RAGT). Subgroup analyses based on the year of publication: late studies published 2015–2020 and early studies 2007–2014. *: assessed during walking at a self-selected velocity SSV; ^: assessed during walking at the fastest velocity possible FV; CI: confidence interval; df: degrees of freedom; SD: standard deviation [file 12984_2021_857_MOESM4_ESM.pdf]

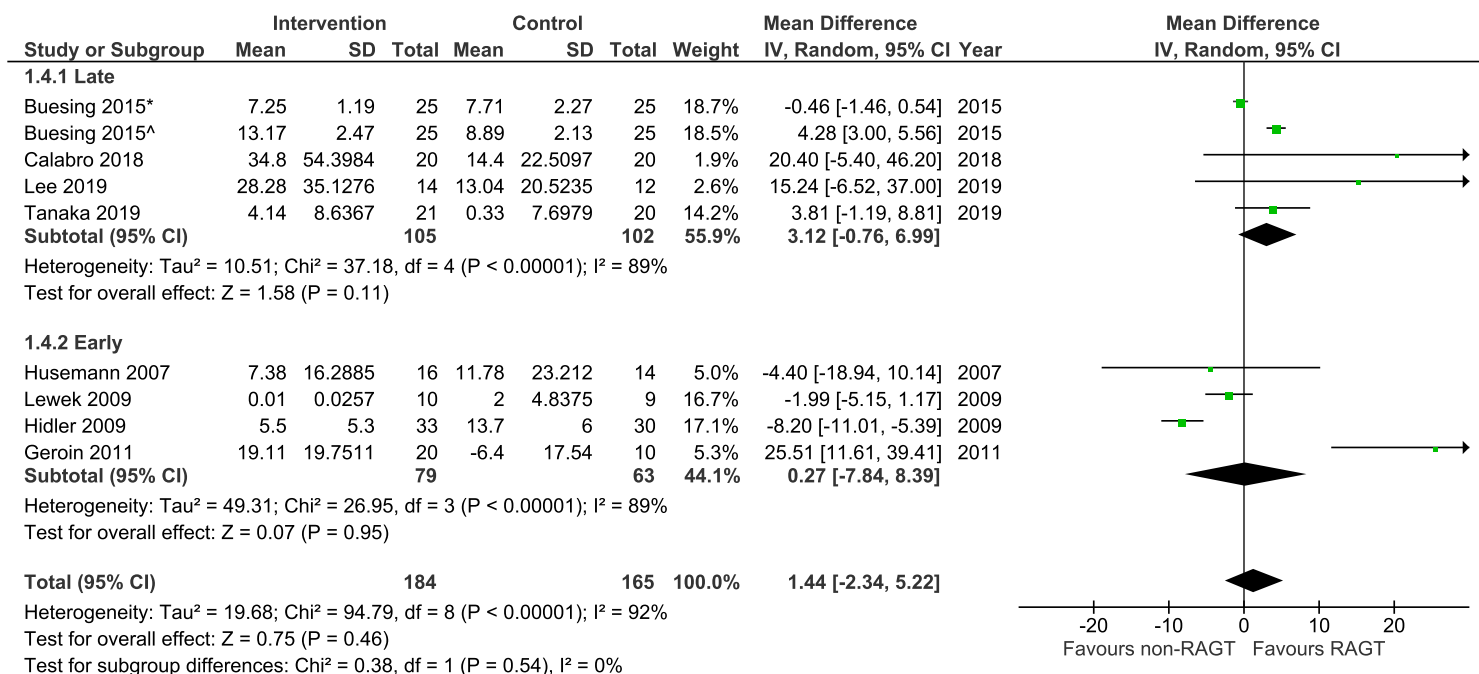

Supplement: Supplementary file 5 — Additional file 5: A forest plot (generated with the Review Manager Web, The Cochrane Collaboration, 2019, available at revman.cochrane.org) summarizing a pooled effect estimate on change in cadence (steps/min), following robotic assisted gait training (RAGT) compared with non-robotic gait training (non-RAGT). Subgroup analyses based on the year of publication: late studies published 2015-2020 and early studies 2007-2014. *: assessed during walking at a self-selected velocity SSV; ^: assessed during walking at the fastest velocity possible FV; CI: confidence interval; df: degrees of freedom; SD: standard deviation [file 12984_2021_857_MOESM5_ESM.pdf]
